# Electroantennographic Responses of Wild and Laboratory-Reared Females of *Xyleborus affinis* Eichhoff and *Xyleborus ferrugineus* (Fabricius) (Coleoptera: Curculionidae: Scolytinae) to Ethanol and Bark Volatiles of Three Host-Plant Species

**DOI:** 10.3390/insects13070655

**Published:** 2022-07-21

**Authors:** Patricia Romero, Luis A. Ibarra-Juárez, Daniel Carrillo, José A. Guerrero-Analco, Paul E. Kendra, Ana L. Kiel-Martínez, Larissa Guillén

**Affiliations:** 1 Red de Manejo Biorracional de Plagas y Vectores, Instituto de Ecología AC (INECOL)—Clúster Científico y Tecnológico BioMimic®, Carretera Antigua a Coatepec 351, El Haya, Xalapa 91073, Veracruz, Mexico; 2 Red de Estudios Moleculares Avanzados, Instituto de Ecología AC (INECOL)—Clúster Científico y Tecnológico BioMimic®, Carretera Antigua a Coatepec 351, El Haya, Xalapa 91073, Veracruz, Mexico; luis.ibarra@inecol.mx (L.A.I.-J.); joseantonio.guerrero@inecol.mx (J.A.G.-A.); ana.kiel@inecol.mx (A.L.K.-M.); 3 Consejo Nacional de Ciencia y Tecnología (Cátedras) Commissioned to Instituto de Ecología AC (INECOL), Xalapa 91073, Veracruz, Mexico; 4 Tropical Research and Education Center, University of Florida, Homestead, FL 33031, USA; dancar@ufl.edu; 5 United States Department of Agriculture, Agricultural Research Service, Subtropical Horticulture Research Station, 13601 Old Cutler Rd., Miami, FL 33158, USA; paul.kendra@usda.gov

**Keywords:** ambrosia beetles, electroantennography (EAG), host kairomones, olfaction, volatilome

## Abstract

**Simple Summary:**

The ambrosia beetles *Xyleborus affinis* and *Xyleborus ferrugineus* are wood borers reported as secondary vectors of pathogenic fungi that cause lethal vascular diseases in mango, cacao, and trees within the laurel family. The use of specific attractants or repellants is one potential method for monitoring or controlling these pests. Chemical ecology studies to develop such tools often use wild or laboratory-reared beetles without first determining whether there are differences in their responses. We compared the antennal olfactory responses of wild and laboratory-reared *X. affinis* and *X. ferrugineus* to bark odors of gumbo-limbo (*Bursera simaruba*), mango (*Mangifera indica*) and chinini (*Persea schiedeana*) with different aging times and used GC–MS to analyze the chemical composition of these bark odors. The antennal responses of laboratory-reared and wild females differed in *X. affinis* and *X. ferrugineus* when interacting with odors. In addition, both beetle species displayed stronger antennal responses to aged bark odors of gumbo-limbo and chinini, apparently due to changes in volatile emissions over time.

**Abstract:**

Chemical ecology studies on ambrosia beetles are typically conducted with either wild or laboratory-reared specimens. Unlike laboratory-reared insects, important aspects that potentially influence behavioral responses, such as age, physiological state, and prior experience are unknown in wild specimens. In this study, we compared the electroantennographic (EAG) responses of laboratory-reared and wild *X. affinis* and *X. ferrugineus* to 70% ethanol and bark odors (host kairomones) of *Bursera simaruba*, *Mangifera indica*, and *Persea schiedeana* aged for 2, 24, and 48 h. Chemical analyses of each odor treatment (bark species x length of aging) were performed to determine their volatilome composition. EAG responses were different between laboratory-reared and wild *X. ferrugineus* when exposed to ethanol, whereas wild *X. affinis* exhibited similar EAG responses to the laboratory-reared insects. Ethanol elicited the strongest olfactory responses in both species. Among the bark-odors, the highest responses were triggered by *B. simaruba* at 48 h in *X. affinis*, and *P. schiedeana* at 24 and 48 h in *X. ferrugineus*. Volatile profiles varied among aged bark samples; 3-carene and limonene were predominant in *B. simaruba*, whereas α-copaene and α-cubebene were abundant in *P. schiedeana.* Further studies are needed to determine the biological function of *B. simaruba* and *P. schiedeana* terpenes on *X. affinis* and *X. ferrugineus,* and their potential application for the development of effective lures.

## 1. Introduction

*Xyleborus affinis* Eichhoff and *Xyleborus ferrugineus* (Fabricius) are two ambrosia beetles (Curculionidae: Scolytinae: Xyleborini) widely distributed in tropical and subtropical areas throughout the world [1,2]. In the Americas, both species are considered secondary pests of economically important plant species, including Spanish cedar (*Cedrela odorata* L.), cacao (*Theobroma cacao* L.), mango (*Mangifera indica* L.), pink poui (*Tabebuia rosea* (Bertol)), tropical plum (*Spondias mombin* L.), gumbo-limbo (*Bursera simaruba* L.), avocado (*Persea americana* Mill.), and sugar cane (*Saccharum officinarum* L.), among others [3,4,5,6]. Like other xyleborine ambrosia beetles, *X. affinis* and *X. ferrugineus* have symbiotic relationships with fungi [7,8], are haplodiploid (i.e., unfertilized eggs produce males and fertilized eggs produce females), exhibit sibling mating, and only females fly to locate and colonize new hosts [8,9,10].

The relationship of these ambrosia beetles with various phytopathogenic fungi has been reported by several authors. For example, *X. affinis* is considered a secondary vector of *Ceratocystis fimbriata* Ellis and Halst, the causal agent of mango wilt [11,12], and *X. ferrugineus* has been reported as one of the main vectors of *Ceratocystis cacaofunesta* Engelbrecht and T.C. Harr., the causal agent of cacao wilt [13,14]. Recently, both beetle species were reported as secondary vectors of *Raffaelea lauricola* T.C. Harr., Fraedrich and Aghayeva (Ophiostomatales: Ophiostomataceae) [6,15,16], the causal agent of lethal laurel wilt in members of the Lauraceae family. Although laurel wilt is not present in Mexico, in recent years, some native species of ambrosia beetles, such as *X. affinis, X. volvulus,* and *X. ferrugineus*, have been reported infesting avocado and mango crops [5,17].

The primary tool for monitoring ambrosia beetle populations consists of lures for host-seeking (i.e., in-flight) females [18,19,20,21]. In the case of *Xyleborus* genus, most species respond to ethanol, a volatile indicative of stressed and dying trees [22,23,24,25]; however, *Xyleborus* attraction to different plant-emitted sesquiterpenes (kairomes) has also been reported [18,26,27,28]. Both *X. affinis* and *X. ferrugineus* showed attraction and olfactory chemoreception (quantified by electroantennography, EAG) to silkbay wood (*Persea humilis* Nash) volatiles, as well as to phoebe and manuka oils with a high terpenoid content [19,29].

Many EAG studies on ambrosia beetles have been performed with wild specimens [18,19,20], field-caught using a baiting method that captures dispersing beetles with host-based attractants [21]. However, factors such as life history, age, mating status, and sexual maturity were unknown in these studies. The availability of wild beetles is an aspect that can limit these types of studies, and that depends on the geographic distribution, population dynamics, climatic conditions, and seasonal presence of the insect species, among other factors. Laboratory-reared insects cultured under controlled conditions (i.e., temperature, humidity, photoperiod, nutrition, etc.) provide a stable supply of insects for experiments [30,31,32]. The physiology of laboratory and wild individuals can vary and these differences can affect the insect’s response to semiochemicals, such as minor antennal depolarizations of laboratory insects [33,34,35,36,37], different host selection responses (i.e., behavioral modifications) [38], and even variations in survival and development of insects [39]. In this research, the main objective was to assess whether there were differences in EAG responses of wild (WF) and laboratory-reared (LRF) *X. affinis* and *X. ferrugineus* females to 70% ethanol and host volatilomes from bark with increasing aging-time, as older samples may mimic the volatile host-location cues emitted from dying or weakened host trees. We also characterized the volatilomes of bark.

## 2. Materials and Methods

### 2.1. Insects

#### 2.1.1. Field Collection

Wild *X. affinis* and *X. ferrugineus* were captured in flight from May–November 2018 and February–March 2019 in Los Tuxtlas, Veracruz, Mexico (18°26′19″ N, 95°11′36″ W, 295 m altitude), using the methodology reported by Kendra et al. [21] with minor modifications. The collection time was 18:00–20:30 h, during the peak flight activity reported for both species [19,40]. Alpha-copaene lures (Synergy Semiochemicals Corp., Burnaby, BC, Canada), and an open plastic container with 50 mL of 70% ethanol were placed at the center of a white blanket on the ground. Additionally, 70% ethanol was sprayed on the blanket at 10 min intervals using a hand-held sprayer. Beetles flying over the area were gently patted towards the blanket, collected with a fine brush, and transferred to Petri dishes with a damp filter paper disc. The beetles were transported to the Chemical Ecology Laboratory of the BioMimic^®^ Scientific and Technological Cluster, INECOL and were kept at controlled conditions (28 ± 1 °C and 68 ± 5% r.h.) until the next day for testing.

#### 2.1.2. Species Identification

Wild insects were identified to the species level following a two-step process. For *X. affinis*, we considered the presence of elytral declivity, which becomes gradually convex, opaque in appearance with the presence of small granules and a yellow to reddish-brown body color. For *X. ferrugineus*, we considered the presence of spines on the elytral declivity and the reddish-brown color of the body [2,41]. Given the similarities between *X. affinis* and *Xyleborus volvulus* Fabricius, and between *X. ferrugineus* and *Xyleborus bispinatus* Eichhoff [42,43], the thorax and abdomen of each female used in the EAG assays were preserved in vials with 70% ethanol. Beheaded specimens were mounted for a species confirmation procedure using the same taxonomic keys as used for the preliminary identification and with comparison to reference individuals previously identified by T.H. Atkinson (Insect Collection, University of Texas, Austin, TX 78703, USA)

#### 2.1.3. Laboratory Rearing

Laboratory-reared *X. affinis* (F.13) and *X. ferrugineus* (F.15) adults were obtained from the Molecular Entomology Laboratory of the BioMimic^®^ Cluster at INECOL and were reared on a culture medium based on sawdust obtained from *Platanus mexicana* Moric [30]. Three and four generations of *X. affinis* and *X. ferrugineus*, respectively, were reared under controlled conditions for use in the EAG experiments. For this, one female was placed in a tube with 15 mL of sterilized and solidified medium that was incubated in an environmental chamber (Thermo Fisher Scientific, 3940, Marietta, OH 45750 USA) at 26 ± 1 °C and 60 ± 5% r.h. and darkness during 30 and 35 days for *X. affinis* and *X. ferrugineus*, respectively. After this time, sclerotized, approximately 1–10-day-old beetles were collected for use in EAG assays (at an interval of approximately 2 h after bioassays).

### 2.2. Bark Volatiles for EAG Tests and Chemical Characterization (CG-MS)

#### 2.2.1. Host Collection

Host plant materials were collected in Xalapa, Veracruz, Mexico (19°30′33′’ N, 96°56′35′’ W; altitude 1241 m). Branches of *B. simaruba* (Bs), *M. indica* (Mi) and *P. schiedeana* (Ps) of 30 cm in length and 6 cm diameter were cut and transported to the laboratory for further processing.

#### 2.2.2. Wood Preparation

Preliminary EAG tests (n = 15) compared the response of both beetle species to pristine wood and grated bark of the three plant species. The bark generated higher responses so that grated bark was chosen as the emitting source of volatiles. For EAG tests and chemical characterization of host-bark volatilomes, branches were gently cleaned with a soft brush to remove mosses, lichens, insects and dust. Cleaned branches were individually processed with a steel grater to obtain grated bark. For the EAG assays, a 15 g sample of grated bark was placed in a 250 mL glass jar and closed with a cap fitted with an airtight nozzle that allowed volatile extraction without opening the jar. For the chemical characterization, 30 g of grated bark were placed in glass jars with lids adapted to collect volatile compounds. For EAG and GC–MS, an empty jar was used as a negative control. A jar with 15 mL of 70% ethanol was used as a positive control for EAG tests. The bark for all treatments was grated on the first day of the experiment. After 2 h (t1), the grated bark was used for EAG tests and for the collection of volatiles. EAG tests and volatile collections were also performed the next day using 24 h-old grated bark samples (t2) and the third day using 48 h-old bark samples (t3). All jars were maintained at 27 ± 1 °C and 60 ± 5% R.H. for 2 h to allow sample volatiles to saturate the headspace. All treatments (bark and controls) were tested and characterized.

### 2.3. Electroantennographic (EAG) Responses

The antennal response was measured by using a Syntech EAG system (Syntech, Kirchzarten, Germany), comprising a micromanipulator (EAG combi probe), a signal connector (IDAC-2), a stimulus controller (CS-55) and the EAG software EAGPro Version 2.0, (Syntech, Kirchzarten, Germany) using the saturated vapor methods reported previously [19]. Wild and laboratory insects were measured separately. A mean (+ S.E.) of 12 ± 2 beetles was tested for each bark aging period (2, 24, and 48 h; n = 36 ± 6) and the entire assay was performed on three different occasions, using a total of 108 ± 18 individuals per origin. The head of each living female was cut with an entomological dissecting scalpel, and in the case of wild individuals, the thorax and abdomen were placed in 70% ethanol and labeled for subsequent species identification. The head was divided into halves with an entomological scalpel. Then, a complete antenna was carefully separated without damaging the basal segment. One antenna was mounted in an electrode holder (Syntech) previously impregnated with a thin layer of Spectra 360 electrode gel (Parker Laboratories, Fairfield, CA, USA). The basal antennal segment was placed on one side of the electrode and the antenna club (the area with the highest sensilla content) was placed facing the injection tower. Subsequently, a filtered airflow at 400 mL/min was run through the injection tube, located 1 mm away from the electrode. When the antenna signal was stable, 4 mL of the headspace, containing volatiles at saturation from one of the treatments, was injected using a pressure-lok^®^ syringe (VICI precision sampling, 050035, Baton Rouge, LA, USA). To obtain the headspace sample, a needle was inserted into the jar through a silicone septum in the airtight nozzle to prevent leaks, and the air was homogenized by pumping with the syringe three times before removing the final injection volume of 4 mL. Each antenna was considered a replicate and each replicate started with the injection of 70% ethanol (positive control), followed by the injection of treatments (wood volatiles) and the negative control (air) in a randomized sequence. Finally, an additional injection of 70% ethanol was administered to evaluate antennal viability. Each consecutive injection was conducted at intervals of two minutes to prevent antennal adaptation, i.e., a decline in olfactory receptor response due to repeated stimulation with odorants.

### 2.4. Characterization of Volatile Organic Compounds (VOCs)

#### 2.4.1. Collection of Bark Volatiles

The dynamic aeration technique was used to collect volatile organic compounds (VOCs) from bark [44]. A 30 g sample of grated bark was placed in a 500 mL glass jar with a modified cap for air inlet and outlet. In the inlet port of the lid, a Teflon tube with a charcoal filter was connected, whereas in the outlet port, a PoraPak-Q filter^®^ (150 mg, 4” Long Porapak-Q^TM^ [20330-U], for solvent extraction P/N: VCT-1/4-3-POR-Q, Gainesville, FL, USA) to retain VOCs was connected to a Teflon tube attached to a vacuum pump. The air injection and vacuum flows were generated with a vacuum pump (Weg N.2536OE1XA56J, Mexico). Purified airflow (1 L/min) was injected into the jar to mix with bark volatiles and then vacuumed to trap them in the PoraPak-Q filter^®^. The volatile collections were carried out under controlled conditions of temperature and humidity (27 ± 1 °C and 60 ± 5% r.h.) for 30 min. At the end of the first VOC collection (2 h-old bark, t1), the PoraPak-Q^®^ filter was removed and the retained volatiles were eluted with 400 µL of methylene chloride (Suprasolv^®^, 99.5% pure, EMD Millipore Corporation, Darmstadt, Germany) stored in a 2 mL amber glass vial, labeled, and stored at −80 °C in a freezer (Thermo Fisher Scientific, FORMA 900 Series, Marrieta, OH, USA) until required for chromatographic analyses. When removing the PoraPak-Q^®^, the system was sealed with Teflon tape (Southland) to prevent the entry of environmental air and the escape of VOCs until the following sample was taken at 24 h (t2). The procedure was repeated for the 48 h (t3) treatment. Three collections of volatiles from grated bark samples and their negative control (empty jar) were made under the same conditions and aging times for each species.

#### 2.4.2. Analyses of VOCs by GC–MS

Bark VOCs were analyzed using a gas chromatograph (GC-2010 Plus, Shimadzu, Kyoto, Japan) coupled to a single quadrupole mass spectrometer (QP-2010 Ultrasystem, Shimadzu, Kyoto, Japan). One microliter of each sample was injected in split mode (16.7 rate) at 250 °C into the GC. Helium gas was used as carrier gas (1.2 mL/min, constant flow) and a ZB-5MSi column (30 m × 0.25 mm ID × 0.25 µm film thickness; Zebron, Phenomenex) was used as a stationary phase. The oven temperature profile started at 50 °C for 4 min, increasing at a rate of 15 °C/min to 250 °C for 5 min. The MS was operated in electron impact (70 eV) mode with an ion source temperature of 230 °C, an interface temperature at 250 °C and a continuous scan from 30 to 500 m/z. Mass spectra data of volatile compounds were compared with those in the NIST/EPA/NIH Mass Spectral Library, NIST 11, Software version 2.0 [National Institute of Standards and Technology, http://www.nist.gov (accessed on 14 July 2022), using a range of 80–99% similarity values as accuracy criterium, with the Labsolutions GCMS solutions 2.72 software, and by co-elution with authentic standards (Sigma–Aldrich Co. LLC., Saint Louis, MO, USA; Toronto Research Chemicals Inc., Toronto, Canada and FUJIFILM Waki Chemicals Corp, Osaka, Japan) under the same analytical conditions as described above. The relative abundances of the putatively annotated VOCs were expressed as adjusted peak area and corrected considering the area of the compounds detected in the controls. The analyses of VOCs were carried out in triplicate.

### 2.5. Data Analyses

The absolute values of each EAG depolarization peak (mV) were used. From each replicate, the value corresponding to its negative control (air) was subtracted to remove the effect of the mechanical movement produced by the injection. When the air values exceeded the treatment values, a non-response was recorded and the observation was discarded. Normality and homoscedasticity tests of EAG responses detected variance heterogeneity and non-normality, so data were log+1 transformed to normalize residuals. A generalized linear model (GLM: likelihood-ratio X^2^ and ANOVA Type III) analysis with Gaussian distribution and link function “identity” was used to determine if the EAG responses of LRF and WF of *X. affinis* and *X. ferrugineus* varied according to bark volatiles of Bs, Mi and Ps bark with the three aging times (2, 24, and 48 h) and the control (70% ethanol). A posthoc test was applied for correction of pairwise comparisons (function “emmeans”, package emmeans) [45]. Data exploration and GLM modeling were performed with the Tlamatini package version 0.2 [46]. The relative abundance of compounds identified in the host-bark volatilomes, were compared by one-way analysis of variance (ANOVA) with a Tukey *post-hoc* test for comparisons of means. All the analyses were performed using R Studio, Inc. version 4.1.2. [47].

## 3. Results

### 3.1. EAG responses of Wild and Laboratory-Reared Insects

**Figure 1:**
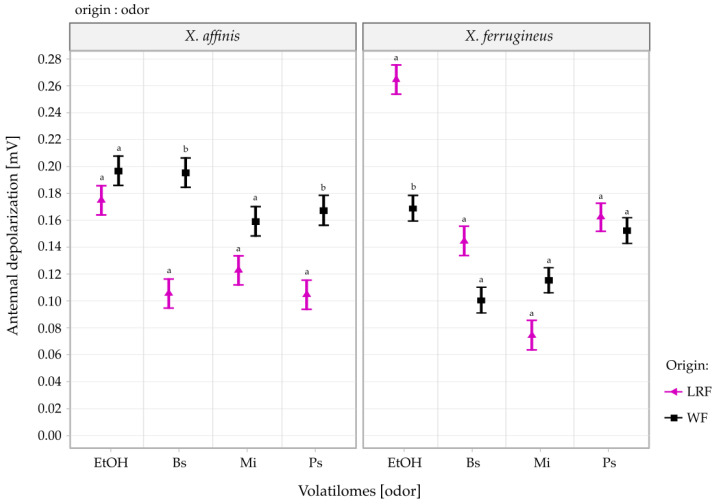
Mean (±SE) EAG responses of two ambrosia beetle species to 70% ethanol (EtOH) and bark volatilomes of Bs (*Bursera simaruba*), Mi (*Mangifera indica*), and Ps (*Persea schiedeana*). Different letters indicate significant differences in contrast tests between the responses to volatilomes from laboratory-reared females (LRF) and wild females (WF) (*p* < 0.05).

Table 1 shows the effects on the antennal response of the main factors and their interactions. In *X. affinis,* the insect origin and odor (volatilomes used as stimuli) treatments generated different EAG responses (Figure 1), but EAG responses were not affected by the aging time of the volatilomes in this species. In *X. ferrugineus*, changes in the responses were not linked to the origin of the beetles, whereas odor and aging time significantly affected antennal responses. However, the only odor that caused a significant difference in responses between wild and laboratory-reared females was ethanol 70% (Figure 1).

For both ambrosia beetle species, the “origin:odor” interaction was significant (Table 1). The graphical representation of these interactions and the contrast tests are presented in Figure 1. In both *X. affinis* and *X. ferrugineus*, 70% ethanol elicited the strongest antennal responses. In *X. ferrugineus*, bark volatilomes generated similar responses, whereas in *X. affinis*, the magnitude of the antennal response varied according to the plant species used (Figure 1), in that both Bs (t = −5.70, df = 565, *p* < 0.001) and Ps (t = −4.00, df = 565, *p* = 0.001) triggered different responses between WF and LRF. In *X. ferrugineus*, the LRF tended to have higher antennal responses than the WF, except for those exposed to Mi bark. However, in this beetle, the only difference found in the contrast tests corresponds to 70% ethanol (t = 6.64, df = 596, *p* < 0.001), although it is important to note that Bs was borderline significant (t = 3.00, df = 596, *p* = 0.055).

### 3.2. EAG Responses of LRF and WF to Different Volatilomes

**Figure 2:**
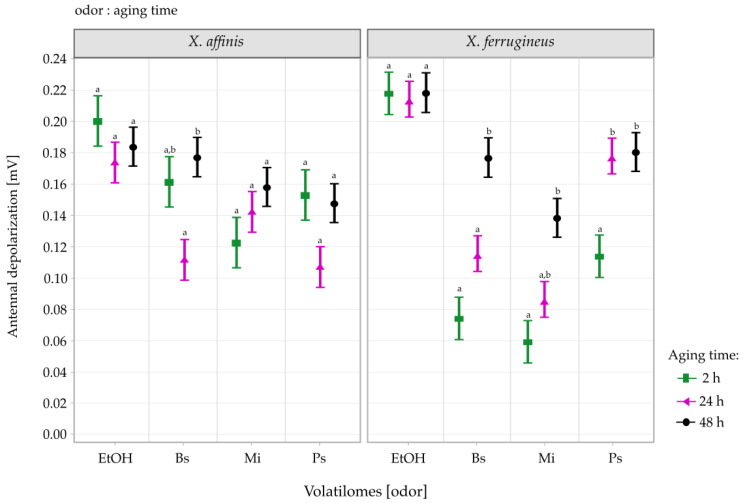
Mean (±SE) EAG responses of *X. affinis* and *X. ferrugineus* (both: wild and laboratory-reared beetles) to 70% ethanol (EtOH) and bark volatiles of Bs (*Bursera simaruba*), Mi (*Mangifera indica*), and Ps (*Persea schiedeana*) at 2, 24, and 48 h after collection of bark samples (aging time). Different letters above each bark treatment indicate significant differences among aging times (*p* < 0.05).

The interaction of the odor:aging time factors was not significant for *X. affinis* (Table 1). The aging time main effect was not significant, and the comparison of Bs at 24 h and 48 h of aging time was the only significant contrast (t = −3.570, df = 565, *p* = 0.019; Figure 2).

In *X. ferrugineus*, the main effect of aging time was significant as well as its interaction with volatilomes (Table 1). The response of *X. ferrugineus* tended to increase with volatilome aging time (Figure 2). The Bs contrast test indicated that the EAG responses at 2 h (2 h vs. 48 h: t = −5.50, df = 596, *p* < 0.001) and 24 h (24 h vs. 48 h: t = −3.61, df = 596, *p* = 0.016) were significantly lower than those recorded at 48 h. A similar trend was observed in Mi where the highest antennal depolarizations were recorded at 48 h and were significantly different from the lowest depolarizations recorded at 2 h-aging (2 h vs. 48 h: t = −4.26, df = 596, *p* = 0.001). Finally, in Ps the highest antennal responses were triggered at 24 h and 48 h, which were similar to one another, and both differed significantly from the 2 h sample (2 h vs. 24 h: t = −3.63, df = 596, *p* = 0.015; and 2 h vs. 48 h: t = −3.60, df = 596, *p* = 0. 017).

### 3.3. Characterization of Volatile Organic Compounds (VOCs) from Grated Bark

Most of the VOCs identified in the bark volatilomes were terpenes (Table 2). The volatilome composition was qualitatively and semiquantitatively different among bark types and aging times. For example, the volatilome of Mi bark presented the simplest composition with a total of six VOCs, whereas the chemical profiles of Bs and Ps were similar in the number of VOCs with 20 and 21, respectively. The volatilome appears to change over time, as some VOCs were present or absent depending on the aging time, the ANOVA and Tukey comparisons are reported in Table 3.

For Bs, the terpenes with the highest percentage of relative abundance (%RA) in the 2 h samples were: 3-carene (48.54%) and limonene (27.56%) (Table 2); however, there was a slight increase in 3-carene emissions at 24 h (52.58%), a percentage that remained almost unchanged at 48 h (52.22%). In the case of limonene, changes in the abundance were also observed over aging times that varied significantly in Ps but not in Bs.

In the Mi bark, most VOCs were only emitted in the 2 h sample, with the highest relative abundances recorded for hexanal (21.15%) and an unknown compound (Unknown 6 in Table 2). The Unknown 6 compound increased in relative abundance from 12.16% at 2 h to 46.72% at 24 h, but then drastically decreased at 48 h (Table 2 and Table 3).

The Ps volatilomes at 2 h and 24 h were similar in the number of compounds, 19 and 20, respectively, in contrast with the number of VOCs in the 48 h samples (eight compounds). The most abundant compounds at 2 h were trans-β-ocimene (19.28%) and cis-β-ocimene (15.63%), whereas at 24 h and 48 h of aging, the most abundant compounds were α-copaene (19.65 and 30.52%, respectively) and α-cubebene (17.89 and 28.19%, respectively) (Table 2 and Table 3).

Alpha-copaene was the only compound found in common among the three bark species; it was present in all three aging times of Ps and Mi, but only at 2 h and 24 h in Bs. Eight compounds (α-pinene, camphene, 3-carene, m-cymene, p-cymene, limonene, α-copaene and caryophyllene) were present in the volatilomes of both Bs and Ps. Of those, only 3-carene was present in Bs and Ps samples at all three aging times (Table 2).

## 4. Discussion

It is frequently assumed that laboratory-reared and wild insects behave identically in response to environmental stimuli, meaning that experiments are performed and important decisions are made based on this premise, which may not be valid. In this study, we used EAG to compare olfactory responses of laboratory-reared and wild females of two *Xyleborus* ambrosia beetle species exposed to odors of 70% ethanol and volatilomes released by the bark of three different host plants aged for different periods of time. The wild females of *X. affinis* had higher antennal depolarizations than the laboratory-reared females. This is consistent with previous studies showing that wild and laboratory-reared insects differ in their behavioral responses [37,48]. Laboratory rearing protocols attempt to mimic the insects’ natural conditions by providing the most beneficial nutrients in an artificial diet and an optimal set of climatic conditions (i.e., temperature and relative humidity). However, artificial diets invariably differ from the natural hosts, which can impact the fitness and performance of insects [49,50]. In the case of *X. affinis,* artificial rearing conditions can modify this insect’s microbiome [51], an important factor given that ambrosia beetles maintain symbiotic relationships with fungi. In other species of insects, changes in the microbiome can affect the resistance to pathogens, nutrition, intra-and inter-specific communication and physiology, among other factors [52]. As such, rearing conditions may potentially affect the antennal responses of *X. affinis*. However, no information is available regarding the effect of nutrition on adult antennal development and the sensitivity of olfactory receptors in ambrosia beetles, an aspect that warrants future investigation.

In *X. ferrugineus,* LRF had significantly higher responses than WF to 70% ethanol (Figure 1). Ambrosia beetles are well known to be highly attracted to ethanol [22,23], and the differences in response may have been related to the contrasting rearing conditions and activity level of the two populations. For example, *X. ferrugineus* WF were collected during flight, an activity that consumes large amounts of energy [53], and moreover females have no access to food during dispersal events. In contrast, LRFs were located inside their galleries, relatively inactive and with ample food available. The antennal responses of *X. ferrugineus* could therefore have been affected by the nutritional reserves and body condition of these insects. Another key factor for WF is their odor experience in the field. In nature, insects must discern among relevant odorants embedded in complex mixtures of non-relevant odors, and when exposed to single odorants that mediate foraging behavior, they may respond less strongly [54]. Field-collected WF were likely to have already experienced exposure to ethanol in various odor blends, which may have activated the neural pathways used to distinguish alcohols in odor mixtures, because in the area where these insects were collected, trees had been felled to widen a rural road. This possibly resulted in a reduced response of females when exposed to ethanol vapor.

*Xyleborus affinis* had different antennal responses to the volatilomes of bark treatments. When we compared the interaction of odor:aging time in both *Xyleborus* species, no differences were observed with ethanol, only with the bark volatilomes (Figure 2). Independent of ethanol, the volatilomes that elicited the highest EAG responses were those of Bs at 24 and 48 h in *X. affinis.* In contrast, in *X. ferrugineus* Bs at 48 h, and Ps at 24 h and 48 h generated stronger EAG responses than the other bark treatments (Figure 2). In both beetle species the differences in EAG responses to these treatments could be related to the volatiles emitted by the bark at different aging times.

The compounds identified from bark of the three host species were mainly terpenes (Table 2), which are generally part of the plant primary metabolism or are emitted in response to external factors such as stress, herbivory, and adverse environmental conditions [55,56]. Many of these VOCs have been reported previously as part of the chemical profile of different plant species. The volatilomes of Bs and Ps were the most complex, with at least 20 VOCs (Table 2). Several compounds identified in this study match previous reports for other species of *Bursera* [4,57] and species in the family Lauraceae [27,58]. In the case of Mi, the previously reported VOCs [59,60,61] do not coincide with those identified here, since these authors used different analysis techniques based on tissue extracts. We used fresh and aged grated bark, and in the case of mango (Mi) only six highly volatile compounds were detected in samples taken at 2 h (Table 2).

The sesquiterpene α-copaene was detected in all three hosts evaluated in this study. This compound is emitted by many plant species under normal and stressed conditions, including in the presence of phytopathogens [62]. Interestingly, α-copaene is a primary attractant of *Xyleborus glabratus* Eichhoff, a vector of the laurel wilt pathogen [18,25,29,58,63,64], and in combination with other sesquiterpenes (e.g., α-cubebene, α-humulene, calamenene), appear to comprise a ‘signature laurel bouquet’ used by female *X. glabratus* to locate suitable hosts [18,64]. In our analyses, high levels of α-copaene and α-cubebene were detected from Ps a member of the Lauraceae. The monoterpene α-pinene (emitted by Ps and Bs) is reported to be an attractant for *X. ferrugineus,* and can enhance the attractive effect of ethanol in some ambrosia beetles, including *X. affinis,* although its synergistic effect with ethanol is unclear because it can reduce the captures of some beetle species [65]. Limonene, considered a green leaf volatile (GLV), was present in Bs and Ps and has been reported as a component of attractive volatilomes for other ambrosia beetle species [66,67]. The monoterpene myrcene, found in Ps, has been reported as a repellent for some bark beetles [68], but its relative abundance was low compared to the most abundant VOCs in the Ps bark samples. Terpinolene, a GLV identified in Bs, has been reported as a repellent of different ambrosia beetles [69], but was present at low relative abundance, similar to that of myrcene. The most abundant compounds in the three aging times of the Bs volatilome were 3-carene and limonene. This is in agreement with the volatile profiles reported for other species of Burseraceae [70,71]. The bicyclic monoterpene 3-carene is reported to be an attractant for bark beetles in the genera *Dendroctonus* and *Hylurgops* [72], but at high levels, can act as a repellent and suppress the colonization process of *Dentroctonus pseudotsugae* Hopkins in its primary host plant [73].

Regarding the composition of volatilomes, it is possible that the aged bark samples emitted two types of volatile compounds: constitutive and induced volatiles. In a study comparing volatiles of *Persea* sp. Infected or not with the phytopathogenic fungus *R. lauricola*, the terpenes α-cubebene, eudesmene, and guaiene, also identified in our samples, were exclusive to wood without fungal infection [62], indicating that these would be considered to be constitutive volatile compounds. The induced volatiles are synthesized by altering the usual metabolic pathways and take longer to be released into the environment. Previous studies indicate that the 24 h and 48 h samples could have contained induced volatiles compounds, such as alkenes, carboxylic acids, alcohols, terpenes, and green leaf volatiles [74,75]. We identified aristolene and anisole that are terpenes emitted by plants in response to herbivory and water stress, respectively [76]. The high EAG responses of *X. ferrugineus* and *X. affinis* to bark volatiles at 48 h may have resulted from induced volatiles such as ethanol, associated with decaying wood in dying hosts, which are often attacked by ambrosia beetles [77,78]. The duration of the aging period of the bark resulted in changes in the chemical identity of the volatilomes and also affected the abundance of certain VOCs. For example, the abundance of origanene decreased from 2 h to 48 h, whereas α-cubebene increased proportionally over time. This type of variation has been reported in several plants [79] and is related to inherent plant defense mechanisms against stress generated by abiotic and biotic factors [55,74,80]. Other compounds such as 3-carene (in Bs and Ps) and limonene (in Bs) did not show significant variation over time, which suggests that they are stable compounds or that they are being produced constantly by the bark [81,82]. In general, insects have stronger responses to odor blends than to single compounds [54,64]. Therefore, the EAG responses observed with *X. affinis* and *X. ferrugineus* are likely to be the result of summed receptor potentials to complex mixtures of volatiles presented concurrently to the beetle antennae.

Additional studies using coupled gas chromatography-electroantennographic detection (GC-EAD) would be required to determine the specific volatile components responsible for eliciting antennal responses. The electrophysiology assays only assess olfactory chemoreception, so further research is required to determine the behavioral responses to these compounds [76], including the evaluation of their enantiomers (i.e., molecules that behave like mirror images of each other), since enantiomers can cause different responses in insects [83]. In bark beetles such as *Hylobius abietis* (L.), the neuronal receptors were found to have stereoselectivity to α-pinene and limonene enantiomers [84]. Similarly, in a study with *Sitophilus oryzae* (L.), the (R)-(+)- and (S)-(−)- configurations of five terpenes had different effects on the repellency response of this curculionid [85]. Moreover, the enantiomer (-)-α-copaene can function as an attractant or repellent depending on the *Xyleborus* species [23,65].

## 5. Conclusions

We quantified the EAG responses of wild and laboratory-reared *X. affinis* and *X. ferrugineus* to 70% ethanol and three bark volatilomes (Bs, Mi, and Ps) that had aged for 2, 24, and 48 h. Ethanol vapor triggered the largest amplitude EAG responses in both *X. affinis* and *X. ferrugineus.* The antennal responses of wild *X. affinis* females were stronger than those of laboratory-reared females. Bark volatilomes elicited similar responses in this ambrosia beetle, but Bs at 24 and 48 h elicited the strongest responses. *Xyleborus ferrugineus* wild and laboratory-reared females responded similarly to the odor treatments (alcohol and aged bark). However, both of females had a higher response to aged bark volatiles emitted by Bs at 48 h and Ps at 24 and 48 h. The volatile profiles of Bs, Mi, Ps bark samples differed over time. These changes included (i) presence or absence of specific VOCs, and (ii) an increase or decrease in the relative abundance of certain VOCs. Our findings are consistent with previous research showing that plants can exhibit biochemical changes in response to external factors such as mechanical damage [79,86,87,88,89]. Our findings represent an opportunity to continue researching plant–insect chemical communication focused on developing potential lures for the management or monitoring these ambrosia beetle species.

## Figures and Tables

**Table 1:** Linear mixed models analysis of the effects of origin (wild and laboratory-reared); odor (volatilomes of Bs, Mi Ps and 70% ethanol); aging time (2, 24, and 48 h) treatments on EAG responses of ambrosia beetles.

Species	*X. affinis*			*X. ferrugineus*		
Variables	*X* ^2^	*df*	*p*	*X* ^2^	*df*	*p*
origin	11.30	1	<0.001	2.48	1	0.115
odor	13.58	3	0.003	113.72	3	<0.001
aging time	3.25	2	0.197	28.18	2	<0.001
origin:odor	10.92	3	0.012	47.97	3	<0.001
origin:aging time	27.26	2	<0.001	1.77	2	0.412
odor:aging time	11.08	6	0.086	22.71	6	<0.001

**Table 2:** VOCs in the volatilomes emitted by the bark samples of three species of trees at 2, 24, and 48 h of aging time, analyzed by GC–MS. Relative abundance values followed by different letters (a, b, c) means clear differences among aging times of each compounds within each bark species (*p* < 0.05).

No.	Compound Name		Relative Abundance (%)								
		RT (min)	*Bursera simaruba*			*Mango indica*			*Persea schiedeana*		
			2 h	24 h	48 h	2 h	24 h	48 h	2 h	24 h	48 h
1	hexanal *^1^	4.42				21.151 ± 9.79					
2	3-hexen-1-ol *^1^	5.58				2.972 ± 0.80					
3	1-hexanol *^1^	5.85				4.860 ± 1.59					
4	Anisole *^1^	6.76				3.060 ± 2.71					
5	unknown 1	6.88							0.988 ± 0.43		
6	origanene ^2^	6.93	9.866 ± 1.56 ^a^	6.581 ± 0.63 ^b^	3.444 ± 0.90 ^c^						
7	α-pinene *^3^	7.06	5.528 ± 0.97 ^a,b^	4.244 ± 0.40 ^a^	3.058 ± 0.37 ^a,c^				5.442 ± 2.04 ^a^	2.242 ± 0.63 ^b^	0.00 ^b^
8	camphene *^1^	7.35	0.114 ± 0.04						1.031 ± 0.79 ^a^	0.541 ± 0.26 ^a^	0.00 ^a^
9	sabinene *^1^	7.70	2.369 ± 0.25 ^a^	1.109 ± 0.15 ^b^	0.932 ± 0.11 ^b^						
10	unknown 2	7.75							1.610 ± 0.34 ^a^	0.739 ± 0.13 ^b^	0.00 ^c^
11	β-pinene *^1^	7.79	1.561 ± 0.16 ^a^	1.240 ± 0.09 ^a^	1.310 ± 0.48 ^a^						
12	myrcene *^1^	7.88							7.567 ± 0.12 ^a^	3.984 ± 0.49 ^b^	0.00 ^c^
13	2-carene *^1^	8.10	0.065 ± 0.00								
14	3-carene *^1^	8.25	48.544 ± 0.70 ^a^	52.585 ± 0.61 ^a^	52.221± 9.93 ^a^				7.032 ± 3.04 ^a^	4.027 ± 1.64 ^a^	3.422 ± 1.60 ^a^
15	m-cymene *^1^	8.36	0.343± 0.04						0.283 ± 0.00 ^a^	0.318 ± 0.23 ^a^	0.00 ^b^
16	p-cymene *^1^	8.47	0.685 ± 0.12 ^a^	1.663 ± 0.09 ^b^	2.180 ± 0.08 ^c^				0.560 ± 0.16 ^a^	0.646 ± 0.13 ^a^	0.00 ^b^
17	limonene *^1^	8.49	27.569 ± 1.38 ^a^	29.052 ± 1.34 ^a^	22.844 ± 6.28 ^a^				0.650 ± 0.24 ^a^	0.489 ± 0.10 ^a^	0.00 ^b^
18	trans-β-ocimene *^1^	8.54							19.278 ± 0.31 ^a^	9.912 ± 1.61 ^b^	0.00 ^c^
19	cis-β-ocimene *^1^	8.69							15.629 ± 2.97 ^a^	4.692 ± 0.26 ^b^	0.00 ^c^
20	unknown 3	8.85	0.058 ± 0.01								
21	γ-terpinene *^1^	8.92	0.321± 0.03 ^a^	0.086 ± 0.01 ^a,b^	0.00 ^b,c^						
22	unknown 4	9.23	0.353 ± 0.05 ^a^	0.356 ± 0.03 ^a^	0.00 ^b^						
23	terpinolene *^1^	9.28	1.745 ± 0.23 ^a^	0.678 ± 0.12 ^a^	1.548 ± 2.05 ^a^						
24	α-cubebene *^2^	12.07							9.766 ± 1.65 ^a^	17.887 ± 0.72 ^b^	28.196 ± 2.37 ^c^
25	γlangene ^2^	12.31							2.115 ± 0.32 ^a^	3.852 ± 0.22 ^b^	5.700 ± 0.96 ^c^
26	α-copaene *^2^	12.43	0.052 ± 0.01 ^a^	0.095 ± 0.01 ^b^	0.00 ^c^	6.136 ± 3.18 ^a^	17.484 ± 3.10 ^b^	6.394 ± 3.55 ^a^	11.029 ± 1.02 ^a^	19.654 ± 0.73 ^b^	30.517 ± 2.15 ^c^
27	unknown 5	12.47							5.531 ± 0.69 ^a^	9.485 ± 1.03 ^b^	11.087 ± 0.60 ^b^
28	unknown 6	12.74				12.165 ± 7.21 ^a^	46.729 ± 6.43 ^b^	0.00 ^a^			
29	caryophyllene *^1^	12.87	0.594 ± 0.33 ^a^	1.525 ± 0.33 ^a^	5.276 ± 1.91 ^b^				0.533 ± 0.00 ^a^	0.901 ± 0.12 ^a^	0.00 ^a^
30	Aristolene ^2^	13.02	0.111 ± 0.07 ^a^	0.299 ± 0.10 ^b^	1.259 ± 0.03 ^c^						
31	α-humulene *^1^	13.13								0.428 ± 0.07	
32	humulene *^1^	13.18	0.041 ± 0.00 ^a^	0.092 ± 0.01 ^a^	0.00 ^a^						
33	unknown 7	13.24								1.732± 0.23	
34	unknown 8	13.42							5.175 ± 1.56 ^a^	8.212 ± 1.62 ^a,b^	9.905 ± 2.08 ^b^
35	Guaiene ^2^	13.47							4.487 ± 1.25 ^a^	6.677 ± 1.32 ^a^	7.353 ± 1.7 ^a^
36	Eudesmene ^2^	13.48	0.037 ± 0.01 ^a^	0.059 ± 0.03 ^a^	0.00 ^a^						
37	unknown 9	13.60							2.024 ± 0.13 ^a^	3.858 ± 0.18 ^a,b^	4.701 ± 1.31 ^b^
38	cadinene ^2^	13.65	0.082 ± 0.01 ^a^	0.166 ± 0.00 ^a^	0.00 ^a^						

*^1^ Compounds identified with authentic standards Sigma–Aldrich Co. LLC.; *^2^ Toronto Research Chemicals Inc., *^3^ FUJIFILM Waki Chemicals Corp; ^2^ Compounds identified with the NIST 11 mass spectra library (*p* > 80%).

**Table 3:** One-way ANOVAs comparing the relative abundances of the main volatile compounds emitted by the bark samples of three tree species at three aging times.

*B. simaruba*		*M. indica*		*P. schiedeana*	
Compound	F and *p*-Values	Compound	F and *p*-Values	Compound	F and *p*-Values
α-copaene	F_2,6_ = 148.696; *p* < 0.001	α-copaene	F_2,6_ = 20.285; *p* < 0.01	α-copaene	F_2,6_ = 138.486; *p* < 0.001
α-pinene	F_2,6_ = 11.060; *p* < 0.01	unknown 6	F_2,6_ = 72.242; *p* < 0.001	α-pinene	F_2,6_ = 12.490; *p* < 0.01
3-carene	F_2,6_ = 0.452; *p* = 0.656			camphene	F_2,6_ = 3.691; *p* = 0.09
caryophyllene	F_2,6_ = 14.352; *p* < 0.01			unknown 2	F_2,6_ = 43.778; *p* < 0.01
limonene	F_2,6_ = 2.194; *p* = 0.192			myrcene	F_2,6_ = 513.191; *p* < 0.01
origanene	F_2,6_ = 25.433; *p* < 0.01			3-carene	F_2,6_ = 2.551; *p* = 0.157
sabinene	F_2,6_ = 5.143; *p* < 0.001			m-cymene	F_2,6_ = 10.316; *p* < 0.05
β-pinene	F_2,6_ = 1.714; *p* = 0.257			p-cymene	F_2,6_ =37.000; *p* < 0.001
p-cymene	F_2,6_ = 203.559; *p* < 0.001			limonene	F_2,6_ = 29.961; *p* < 0.001
γ-terpinene	F_2,6_ = 246.840; *p* < 0.001			trans-β-ocimene	F_2,6_ = 312.400; *p* < 0.001
unknown 4	F_2,6_ = 11.519; *p* < 0.001			cis-β-ocimene	F_2,6_ = 65.309; *p* < 0.001
terpinolene	F_2,6_ = 1.344; *p* = 0.329			α-cubebene	F_2,6_ = 86.919; *p* < 0.001
aristolene	F_2,6_ = 217.197; *p* < 0.001			ylangene	F_2,6_ = 47.330; *p* < 0.001
eudesmene	F_2,6_ = 0.268; *p* = 0.773			unknown 5	F_2,6_ = 39.023; *p* < 0.001
cadinene	F_2,6_ = 0.297; *p* = 0.752			caryophyllene	F_2,6_ = 1.283; *p* = 0.343
				unknown 8	F_2,6_ = 5.501; *p* < 0.05
				guaiene	F_2,6_ = 3.243; *p* = 0.110
				unknown 9	F_2,6_ = 9.618; *p* < 0.05

## Data Availability

The data presented in this study are available on request from the corresponding authors.
